# Supplementary material for: The Effect of Resistance Training on Bone Mineral Density in Older Adults: A Systematic Review and Meta-Analysis
Source: Healthcare (Basel). 2022 Jun 17;10(6):1129. doi: 10.3390/healthcare10061129 (PMC9222380; doi:10.3390/healthcare10061129)
Supplement: Supplementary file 1 [file healthcare-10-01129-s001.zip › PEDro_Methodological_Quality_Supplementary File S3.pdf]

Supplementary file #3: The scores of methodological quality for each study from PEDro scale

Study	PEDro ratings								Scores	Classification
	1	2	3	4	8	9	10	11		
Marques et al. [32]	1	1	0	1	1	1	1	1	6	Excellent
Mosti. et al. [4]	00	1	0	1	1	1	1	1	6	Excellent
Bemben & Bemben [31]	0	0	0	1	1	1	1	1	5	Good
Marques et al [11[	1	0	0	1	1	1	1	1	5	Good
Bemben et al [13]	0	0	0	1	1	1	1	1	5	Good
Whiteford et al [12]	1	1	0	1	1	1	1	1	6	Excellent
Bocalini et al {16]	1	1	0	1	1	1	1	1	6	Excellent
