# Supplementary material for: The Effect of Resistance Training on Bone Mineral Density in Older Adults: A Systematic Review and Meta-Analysis
Source: Healthcare (Basel). 2022 Jun 17;10(6):1129. doi: 10.3390/healthcare10061129 (PMC9222380; doi:10.3390/healthcare10061129)
Supplement: Supplementary file 1 [file healthcare-10-01129-s001.zip › Search_PubMed_Supplementary File S2.pdf]

Pubmed Database

Search: (((((((Bone Density) OR Bone mineral content) OR Bone remodeling) AND Bone metabolism) AND Elderly) AND Health) AND Resistance exercise) OR Resistance trainig Filters: Full text, Humans, English, Middle Aged + Aged: 45+ years Filters: in the last 10 years

((("bone density"[MeSH Terms] OR ("bone"[All Fields] AND "density"[All Fields]) OR "bone density"[All Fields] OR ("bone density"[MeSH Terms] OR ("bone"[All Fields] AND "density"[All Fields]) OR "bone density"[All Fields] OR ("bone"[All Fields] AND "mineral"[All Fields] AND "content"[All Fields]) OR "bone mineral content"[All Fields]) OR ("bone remodelling"[All Fields] OR "bone remodeling"[MeSH Terms] OR ("bone"[All Fields] AND "remodeling"[All Fields]) OR "bone remodeling"[All Fields])) AND (("bone and bones"[MeSH Terms] OR ("bone"[All Fields] AND "bones"[All Fields]) OR "bone and bones"[All Fields] OR "bone"[All Fields]) AND ("metabolic"[All Fields] OR "metabolical"[All Fields] OR "metabolically"[All Fields] OR "metabolics"[All Fields] OR "metabolism"[MeSH Terms] OR "metabolism"[All Fields] OR "metabolisms"[All Fields] OR "metabolism"[MeSH Subheading] OR "metabolic networks and pathways"[MeSH Terms] OR ("metabolic"[All Fields] AND "networks"[All Fields] AND "pathways"[All Fields]) OR "metabolic networks and pathways"[All Fields] OR "metabolities"[All Fields] OR "metabolization"[All Fields] OR "metabolize"[All Fields] OR "metabolized"[All Fields] OR "metabolizer"[All Fields] OR "metabolizers"[All Fields] OR "metabolizes"[All Fields] OR "metabolizing"[All Fields])) AND ("aged"[MeSH Terms] OR "aged"[All Fields] OR "elderly"[All Fields] OR "elderlies"[All Fields] OR "elderly s"[All Fields] OR "elderlys"[All Fields]) AND ("health"[MeSH Terms] OR "health"[All Fields] OR "health s"[All Fields] OR "healthful"[All Fields] OR "healthfulness"[All Fields] OR "healths"[All Fields]) AND (("resist"[All Fields] OR "resistance"[All Fields] OR "resistances"[All Fields] OR "resistant"[All Fields] OR "resistants"[All Fields] OR "resisted"[All Fields] OR "resistence"[All Fields] OR "resistences"[All Fields] OR "resistent"[All Fields] OR "resistibility"[All Fields] OR "resisting"[All Fields] OR "resistive"[All Fields] OR "resistively"[All Fields] OR "resistivities"[All Fields] OR "resistivity"[All Fields] OR "resists"[All Fields]) AND ("exercise"[MeSH Terms] OR "exercise"[All Fields] OR "exercises"[All Fields] OR "exercise therapy"[MeSH Terms] OR ("exercise"[All Fields] AND "therapy"[All Fields]) OR "exercise therapy"[All Fields] OR "exercise s"[All Fields] OR "exercised"[All Fields] OR "exerciser"[All Fields] OR "exercisers"[All Fields] OR "exercising"[All Fields]))) OR (("resist"[All Fields] OR "resistance"[All Fields] OR "resistances"[All Fields] OR "resistant"[All Fields] OR "resistants"[All Fields] OR "resisted"[All Fields] OR "resistence"[All Fields] OR "resistences"[All Fields] OR "resistent"[All Fields] OR "resistibility"[All Fields] OR "resisting"[All Fields] OR "resistive"[All Fields] OR "resistively"[All Fields] OR "resistivities"[All Fields] OR "resistivity"[All Fields] OR "resists"[All Fields]) AND "trainig"[All Fields] AND ("filter"[All Fields] OR "filter s"[All Fields] OR "filtered"[All Fields] OR "filtering"[All Fields] OR "filterings"[All Fields] OR "filters"[All Fields]) AND "Full"[All Fields] AND ("text messaging"[MeSH Terms] OR ("text"[All Fields] AND "messaging"[All Fields]) OR "text messaging"[All Fields] OR "text"[All Fields]) AND ("human s"[All Fields] OR "humans"[MeSH Terms] OR "humans"[All Fields] OR "human"[All Fields]) AND "English"[All Fields] AND ("middle aged"[MeSH Terms] OR ("middle"[All Fields] AND "aged"[All Fields]) OR "middle aged"[All Fields]) AND ("aged"[MeSH Terms] OR "aged"[All Fields]) AND "45"[All Fields] AND "years"[All Fields])) AND (y_10[Filter])

Translations

Bone Density: "bone density"[MeSH Terms] OR ("bone"[All Fields] AND "density"[All Fields]) OR "bone density"[All Fields]

Bone mineral content: "bone density"[MeSH Terms] OR ("bone"[All Fields] AND "density"[All Fields]) OR "bone density"[All Fields] OR ("bone"[All Fields] AND "mineral"[All Fields] AND "content"[All Fields]) OR "bone mineral content"[All Fields]

Bone remodeling: "bone remodelling"[All Fields] OR "bone remodeling"[MeSH Terms] OR ("bone"[All Fields] AND "remodeling"[All Fields]) OR "bone remodeling"[All Fields]

Bone: "bone and bones"[MeSH Terms] OR ("bone"[All Fields] AND "bones"[All Fields]) OR "bone and bones"[All Fields] OR "bone"[All Fields]

metabolism: "metabolic"[All Fields] OR "metabolical"[All Fields] OR "metabolically"[All Fields] OR "metabolics"[All Fields] OR "metabolism"[MeSH Terms] OR "metabolism"[All Fields] OR "metabolisms"[All Fields] OR "metabolism"[Subheading] OR "metabolic networks and pathways"[MeSH Terms] OR ("metabolic"[All Fields] AND "networks"[All Fields] AND "pathways"[All Fields]) OR "metabolic networks and pathways"[All Fields] OR "metabolities"[All Fields] OR "metabolization"[All Fields] OR "metabolize"[All Fields] OR "metabolized"[All Fields] OR "metabolizer"[All Fields] OR "metabolizers"[All Fields] OR "metabolizes"[All Fields] OR "metabolizing"[All Fields]

Elderly: "aged"[MeSH Terms] OR "aged"[All Fields] OR "elderly"[All Fields] OR "elderlies"[All Fields] OR "elderly's"[All Fields] OR "elderlys"[All Fields]

Health: "health"[MeSH Terms] OR "health"[All Fields] OR "health's"[All Fields] OR "healthful"[All Fields] OR "healthfulness"[All Fields] OR "healths"[All Fields]

Resistance: "resist"[All Fields] OR "resistance"[All Fields] OR "resistances"[All Fields] OR "resistant"[All Fields] OR "resistants"[All Fields] OR "resisted"[All Fields] OR "resistence"[All Fields] OR "resistences"[All Fields] OR "resistent"[All Fields] OR "resistibility"[All Fields] OR "resisting"[All Fields] OR "resistive"[All Fields] OR "resistively"[All Fields] OR "resistivities"[All Fields] OR "resistivity"[All Fields] OR "resists"[All Fields]

exercise: "exercise"[MeSH Terms] OR "exercise"[All Fields] OR "exercises"[All Fields] OR "exercise therapy"[MeSH Terms] OR ("exercise"[All Fields] AND "therapy"[All Fields]) OR "exercise therapy"[All Fields] OR "exercise's"[All Fields] OR "exercised"[All Fields] OR "exerciser"[All Fields] OR "exercisers"[All Fields] OR "exercising"[All Fields]

Resistance: "resist"[All Fields] OR "resistance"[All Fields] OR "resistances"[All Fields] OR "resistant"[All Fields] OR "resistants"[All Fields] OR "resisted"[All Fields] OR "resistence"[All Fields] OR "resistences"[All Fields] OR "resistent"[All Fields] OR "resistibility"[All Fields] OR "resisting"[All Fields] OR "resistive"[All Fields] OR "resistively"[All Fields] OR "resistivities"[All Fields] OR "resistivity"[All Fields] OR "resists"[All Fields]

Filters:: "filter"[All Fields] OR "filter's"[All Fields] OR "filtered"[All Fields] OR "filtering"[All Fields] OR "filterings"[All Fields] OR "filters"[All Fields]

text,: "text messaging"[MeSH Terms] OR ("text"[All Fields] AND "messaging"[All Fields]) OR "text messaging"[All Fields] OR "text"[All Fields]

Humans,: "human's"[All Fields] OR "humans"[MeSH Terms] OR "humans"[All Fields] OR "human"[All Fields]

Middle Aged: "middle aged"[MeSH Terms] OR ("middle"[All Fields] AND "aged"[All Fields]) OR "middle aged"[All Fields]

Aged:: "aged"[MeSH Terms] OR "aged"[All Fields]
